# Researches into the Structure and Physiology of the Kidney

**Published:** 1857-09

**Authors:** 


ART. VIII.—*Researches into the Structure and Physiology of the Kidney.* By C. E. Isaacs, M. D., Demonstrator of Anatomy in the University of the city of New York. Read March 5, 1856. Published in the Transactions of the New York Academy of Medicine, vol. i, part ix. S. S. & W. Wood. 1857.

It is rare that the journalist in this country has an occasion to notice a paper of this character; a paper giving the results of extended and carefully conducted researches into a province of minute anatomy. The subject is an important one. Points of pathological interest of direct practical application, concern the structure and physiology of the kidney. The investigations of Bowman, and more recently, the elaborate researches of Dr. George Johnson, have shed much light on the subject. Still, there remained doubtful and mooted questions; and the object of Dr. Isaacs in giving his studies this direction was, if possible, to clear up certain obscure and questionable points, and, at the same time, either to correct or confirm those of the statements of other observers which have been considered as established. The paper read to the Academy of Medicine, contains an account of observations continued daily for twelve months. The kidneys of a great number of animals were subjected to microscopical examination, viz., the frog, turtle, snake, alligator, fish, bird, mouse, rat, squirrel, cat, dog, racoon, rabbit, hog, sheep, deer, elk, moose, ox, horse, black bear, rhinoceros, monkey and man. The author says, "Entering upon this examination without any preconceived theory, it has occupied my attention for some years past, and for the last twelve months I have labored on the subject almost daily, sparing neither trouble, time, nor expense, and carefully guarding against arriving at any conclusion until after long-continued and repeated examinations."

In some important conclusions Dr. Isaacs differs from previous observers. Mr. Bowman and Dr. Geo. Johnson regard the epithelium lining the tubes of the of the cortical portion of the kidney, as belonging to the spheroidal or "glandular" variety. Dr. Isaacs is satisfied that these tubes are lined by permanent or tesselated epithelium. He thinks these observers have been deceived by the specimens not being fresh, or in a pathological condition, or the epithelium having undergone change by the mode of examination. On the supposed character of these cells is based, in fact, the opinion of Bowman and Johnson that the true secretion of urine, i. e. the separation of the urinary principles, takes place exclusively in these tubes. Dr. Isaacs, as will be presently seen, dissents from this opinion.*

Bowman and Johnson deny the existence of nucleated cells upon the malpighian tufts: in other words, within the expanded extremities of the convoluted tubes. Dr. Isaacs believes that he has demonstrated the presence of cells in this situation. According to the views of Bowman, the vessels forming the malpighian tuft lying naked within the extremities of the tubes, water transudes readily under the hydraulic pressure of the blood in the arteries; hence, the water of the urine, its escape being due purely to physical causes. If, however, the observations of Dr. Isaacs are correct, the urinary secretion proper is not alone from the renal portal veins, but also from the malpighian tufts; in other words, it takes place into the extremities of the convoluted tubes, as well as along their course. The latter view Dr. Isaacs sustains by various contingent facts.

The existence of a fibrous matrix in the kidney, has been denied by some and maintained by others. Dr. Isaacs appears to have established the fact of its existence beyond a doubt. In demonstrating this, as well as other points by means of the microscope, Dr. Isaacs has resorted to methods of

---

* That is, these observers claim that the secretion takes place within the tubes exclusive of the flasked extremities containing the malpighian tufts.

displaying the structure of the kidney more successful than those hitherto employed. In this consists an original feature of these researches.

The paper contains a great number of wood cuts representing the appearances of the different structural elements of the kidney, in different animals, as seen with the microscope.

We trust that hereafter the medical journalist will have more frequent occasions than heretofore to notice researches of this character. In such scientific contributions we are behind other countries. But with men like Dalton, Isaacs, Leidly, Da Costa and others to lead the way, we may hope that ere long our country will not be open to this reproach. Aside from the intrinsic value of these researches, they will be useful by way of inciting others to similar efforts to advance our knowledge in medical science. They reflect honor on Dr. Isaacs, on the New York Academy of Medicine, and on the profession of this country. *

